# Chronic relapsing inflammatory optic neuropathy

**DOI:** 10.4103/0972-2327.61280

**Published:** 2010

**Authors:** Monica Saini, Dheraj Khurana

**Affiliations:** Department of Medicine, Division of Neurology, Stroke Programme, University of Alberta, Canada; 1 Department of Neurology, PGIMER, Chandigarh, India

**Keywords:** Recurrent optic neuritis, steroid responsive

## Abstract

Chronic relapsing inflammatory optic neuropathy (CRION) is a recently described recurrent optic neuropathy which is steroid responsive. Several features distinguish this entity from optic neuritis associated with demyelinating disorders and connective tissue diseases. The severe degree of visual loss, persistence of pain after onset of visual loss, and recurrent episodes are unique to this disorder. We describe here a patient who presented with recurrent episodes of painful visual loss, followed by resolution of deficits, over a period of ten years. He was diagnosed as isolated optic neuritis conforming to features of CRION.

## Introduction

The syndrome comprising subacute visual loss, pain, and a clear and early response to systemic steroids is easily identifiable as an inflammatory optic neuropathy. Optic neuritis may occur as amanifestation of systemic autoimmune diseases, sarcoidosis, or central nervous system (CNS) demyelinating diseases. Chronic relapsing inflammatory optic neuropathy (CRION) is a recently described form of isolated recurrent optic neuropathy.[1] We describe here a patient with recurrent episodes of optic neuropathy over a period several years, withfeatures conforming to the diagnosis of CRION.

## Case Report

A 26-year-old male presented in June 2006 with complaints of dull ocular pain, decrease in vision over 8–10 days, and painful eye movements in right eye. The pain persisted after the onset of visual loss. On probing, patient gave a history of 16–17 episodes of similar attacks occuring unilaterally over the past ten years, with a single episode involving bilateral eyes sequentially over two weeks. Each episode began with local pain increasing on eye movement and visual loss over 10–12 days. The documented visual acuity during the episodes varied from finger counting at 1 meter toonly perception of light. The interval in between episodes varied from three months to three years. Each episode remitted with systemic steroids and three episodes were temporally related to steroid withdrawal. There was no history suggestive of connective tissue disease or sarcoidosis.

**Figure 1:**
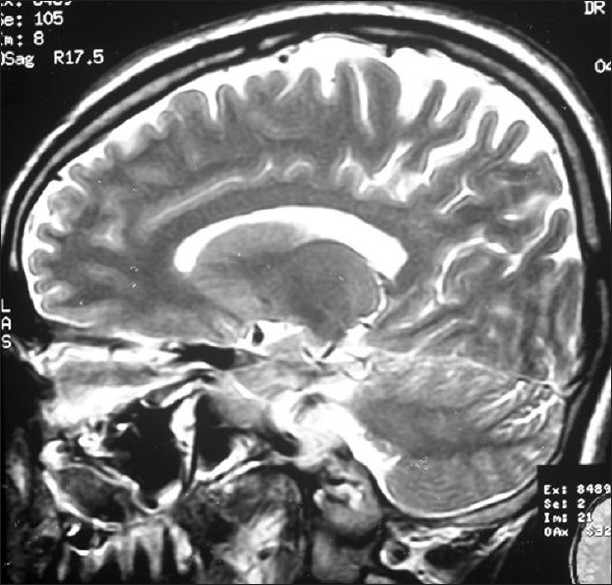
Normal T2 weighted saggital MRI brain in 2003

**Figure 2:**
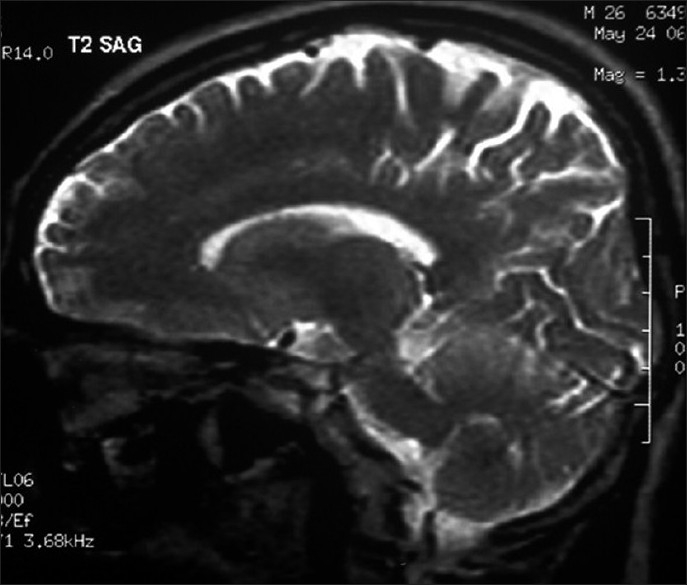
Normal T2 weighted saggital MRI brain in 2006

**Figure 3:**
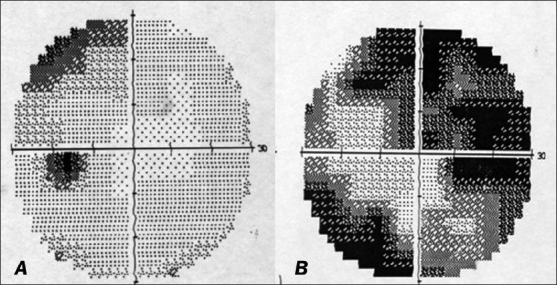
Automated perimetry at index event showing field defects in the left (A) and right (B) eyes

**Figure 4:**
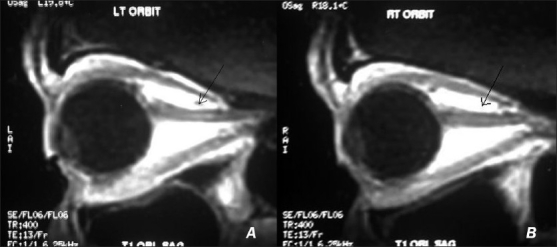
Contrast enhanced MRI showing left (A) and right (B) optic nerves at index event

On examination, patient was able to count fingers at one meter with the right eye, and visual acuity in the left eye was 6/12. Right pupil was reacting sluggishly to light with a relative afferent papillary defect (RAPD). Fundus examination revealed bilateral pale discs. Remaining neurological examination was unremarkable. Perimetry revealed constriction of the visual field in the righteye, without any specific pattern. Laboratory investigations showed a normal erythrocyte sedimentation rate (ESR) and albumin: globulin ratio. The chest X-ray was normal. The Mantoux test and the autoimmune profile (LE cell, antinuclear antibody, and rheumatoid factor) were negative. The Cerebrospinal fluid (CSF) examination was normal and there was no evidence of oligoclonal bands (OCBs) in serum or CSF. Visual evoked responses revealed an increased P100 latency in the right eye (114 m sec) and were normal in the left eye. The brainstem auditory evoked response (BAER) and MRI of the brain and optic nerves (gadolinium enhanced) were normal [Figures 1–4]. Previous investigations including chest x-ray and MRI brain (performed in 2003) were normal. Perimetry performed in previous three episodes showed variable field loss, which did not conform to any specific pattern.

Patient received intravenous methylprednisolone, followed by tapering doses of oral steroids. Pain subsided within two days and vision improved over two weeks to 6/12 in the right eye. At present, patient is maintained on oral deflazacort (1 mg on alternate days), and has been asymptomatic since June 2006.

## Discussion

CRION is a recurrent, steroid dependent optic neuritis, without evidence of any additional neurological deficits, sarcoidosis or systemic autoimmune diseases. Kidd *et al*., in their report of 15 patients, described a syndromic presentation of subacute optic neuropathy, prominent pain, prompt response to systemic corticosteroids, and relapse on steroid withdrawal.[1] These patients had involvement of both optic nerves with the latency (between attacks), varying from days to 14 years. The features that differentiated this group from the classical demyelinating optic neuritis, included persistence of pain after onset of visual loss, the higher degree of visual loss, and frequent relapses. None of these patients had OCBs in the CSF and their MRI of the brain was normal. Out of 30 optic nerves, 19 imaged in these 15 patients showed abnormalities on MRI including thickening, high signal intensity, or enhancement.[1]

Myers *et al.* have described corticosteroid dependent optic neuritis, not associated with demyelinating disease.[2] In their report of 48 patients, 32 had evidence of SLE, sarcoidosis, or other systemic autoimmune disease. Our patient had multiple attacks of steroid responsive painful optic neuropathy, each associated with significant visual loss, over a period of ten years. He has not developed any symptom suggestive of sarcoidosis or other systemic autoimmune disease, and the laboratory investigations for these entities were also negative.

The clinical presentation of our patient was similar to those described by Kidd *et al*., in terms of recurrent episodes of subacute onset of visual defect, significant pain and an excellent response to oral steroids.[1] In individuals with the first episode of isolated optic neuritis (ON), clinical features such as, greater degree of visual loss, prominent pain that persists following the acute phase, and a total resolution of symptoms with steroids may be indicative of a benign course. However, a complete diagnostic work-up for demyelination, autoimmune disease, and other possible causes is mandatory in these patients. The possibility of CRION should be considered only in patients with recurrent and isolated ON. Although, no specific laboratory or clinical criteria have been described for the diagnosis of CRION, clinical indicators, including higher degree of visual loss, persistence of pain after visual loss is established, and relapse on steroid withdrawal is helpful. CRION should be alluded to only after exclusion of demyelination and other autoimmune disorders.

The importance of identifying these patients has therapeutic implication as CRION is highly responsive to steroids and may mandate long-term steroid use to maintain remission. Till date, there are no systematic studies evaluating the duration and intensity of immunosuppression required in patients with CRION; the data is purely observational, based on personal experience. The experiencein our patient indicates that long-term and low dose corticosteroids are an effective and safe option.
